# Supplementary figures and images for: Precise coordination between nutrient transporters ensures fertility in the malaria mosquito Anopheles gambiae
Source: PLoS Genet. 2024 Jan 29;20(1):e1011145. doi: 10.1371/journal.pgen.1011145 (PMC10852252; doi:10.1371/journal.pgen.1011145)

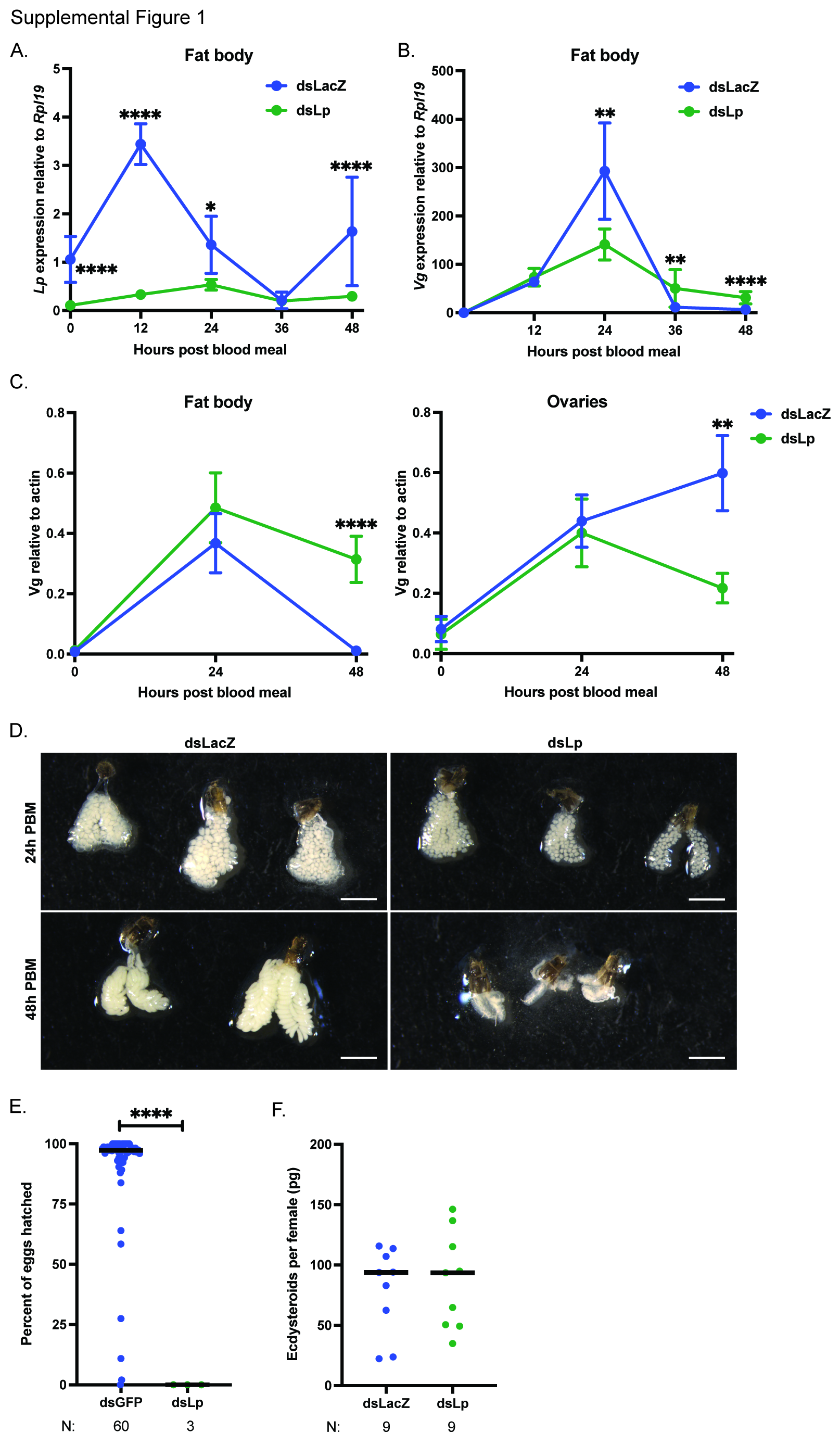

Supplement: S1 Fig — (A) Successful Lp knockdown as determined by RT-qPCR of Lp expression levels relative to Rpl19 in the fat body of dsLacZ and dsLp females; samples of ten tissues, three biological replicates (REML variance component analysis: * = p < 0.05; **** = p < 0.0001; three biological replicates). (B) RT-qPCR of Vg expression levels relative to Rpl19 in the fat body of dsLacZ and dsLp females; samples of ten tissues, four biological replicates (REML variance component analysis: ** = p < 0.01; **** = p < 0.0001; four biological replicates). (C) Western blot quantification from Fig 1C showing an accumulation of Vg in the fat body and a decrease of Vg in the ovaries upon Lp knockdown; samples of five tissues, three biological replicates (REML variance component analysis: ** = p < 0.01; **** = p < 0.0001). (D) Images of ovaries at 24 and 48h post blood meal showing that Lp depleted ovaries develop normally at first before degenerating by 48h; representative images, three biological replicates; scale bar = 2 mm. (E) Lp knockdown causes complete infertility of mated females that lay eggs; each dot represents percent hatch rate per female; N = number of females, pooled from two biological replicates (Mann-Whitney: **** = p < 0.0001). Very few (3 of 71) mated blood fed Lp-knockdown females laid eggs, unlike controls (60 of 64). (F) There is no difference in ecdysteroid levels of whole female bodies at 26h PBM upon Lp knockdown; each dot represents ecdysteroid level per female derived from a sample of 10 females (Unpaired t-test: not significant; three biological replicates of three samples each). (TIF) [file pgen.1011145.s001.tif]

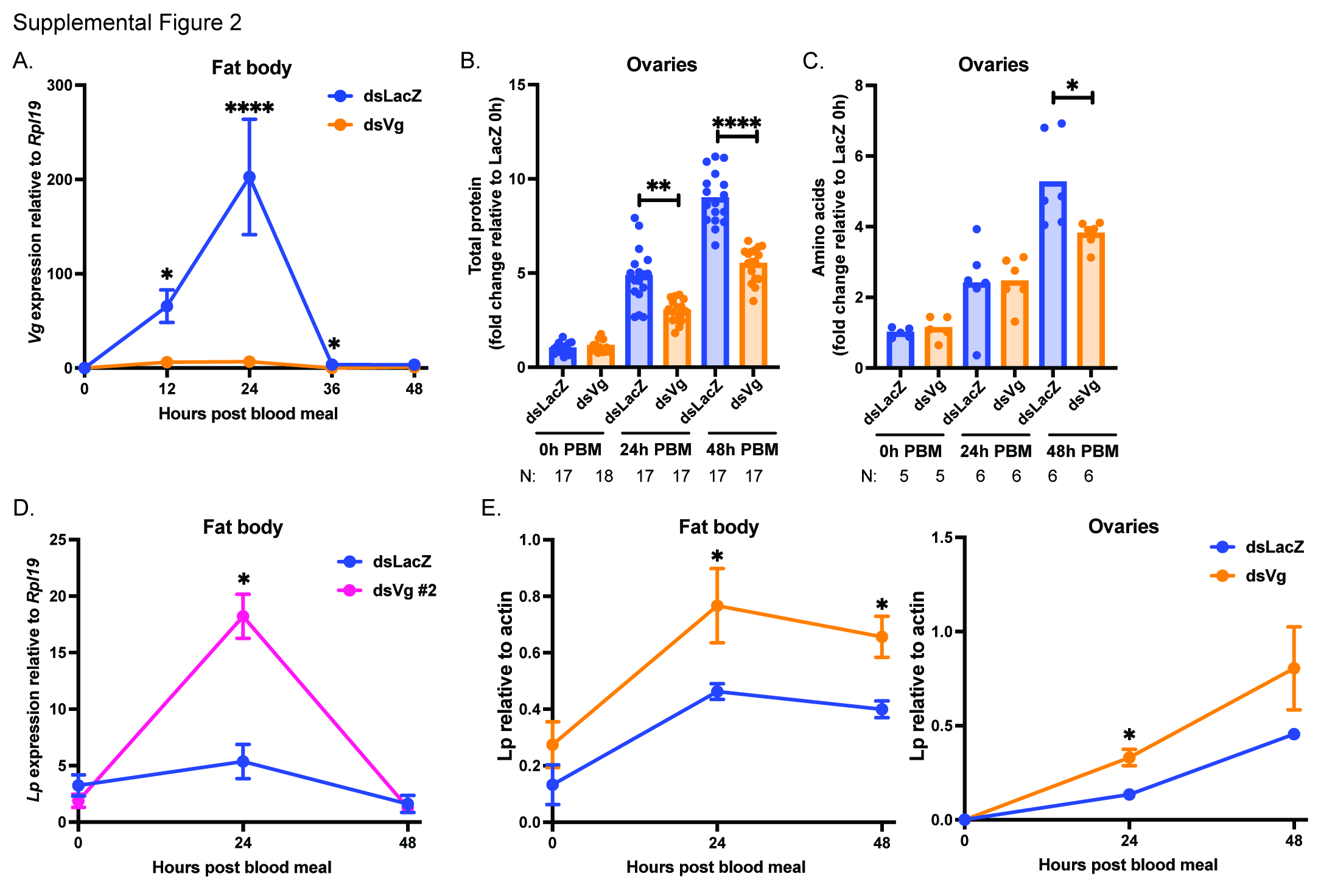

Supplement: S2 Fig — (A) Successful Vg knockdown as determined by RT-qPCR of Vg expression levels relative to Rpl19 in the fat body; samples of ten tissues, three biological replicates (REML variance component analysis: * = p < 0.05; **** = p < 0.0001). (B) Fold change in protein levels measured by Bradford assay in the ovaries of dsLacZ and dsVg females before blood meal and at 24h and 48h post blood meal (PBM); each dot is representative of three pairs of ovaries; N = number of samples of three tissues, pooled from three biological replicates (REML variance component analysis by timepoint: ** = p < 0.01; **** = p < 0.0001). (C) Fold change in free amino acid levels in the ovaries of dsLacZ and dsVg females before blood meal and at 24h and 48h PBM; each dot is representative of five pairs of ovaries; N = number of samples of five tissues, pooled from three biological replicates (REML variance component analysis by timepoint: * = p < 0.05). (D) Vg knockdown by a second Vg RNAi fragment also results in an increase in Lp levels as determined by RT-qPCR; samples of ten tissues, three biological replicates (REML variance component analysis: * = p < 0.05). (E) Western blot quantification from Fig 2G showing an accumulation of Lp in the fat body and ovaries upon Vg knockdown; samples of five tissues, three biological replicates (REML variance component analysis: fat body–dsRNA: p < 0.01; * = p < 0.05; ovaries–dsRNA: p < 0.05; * = p < 0.05). (TIF) [file pgen.1011145.s002.tif]

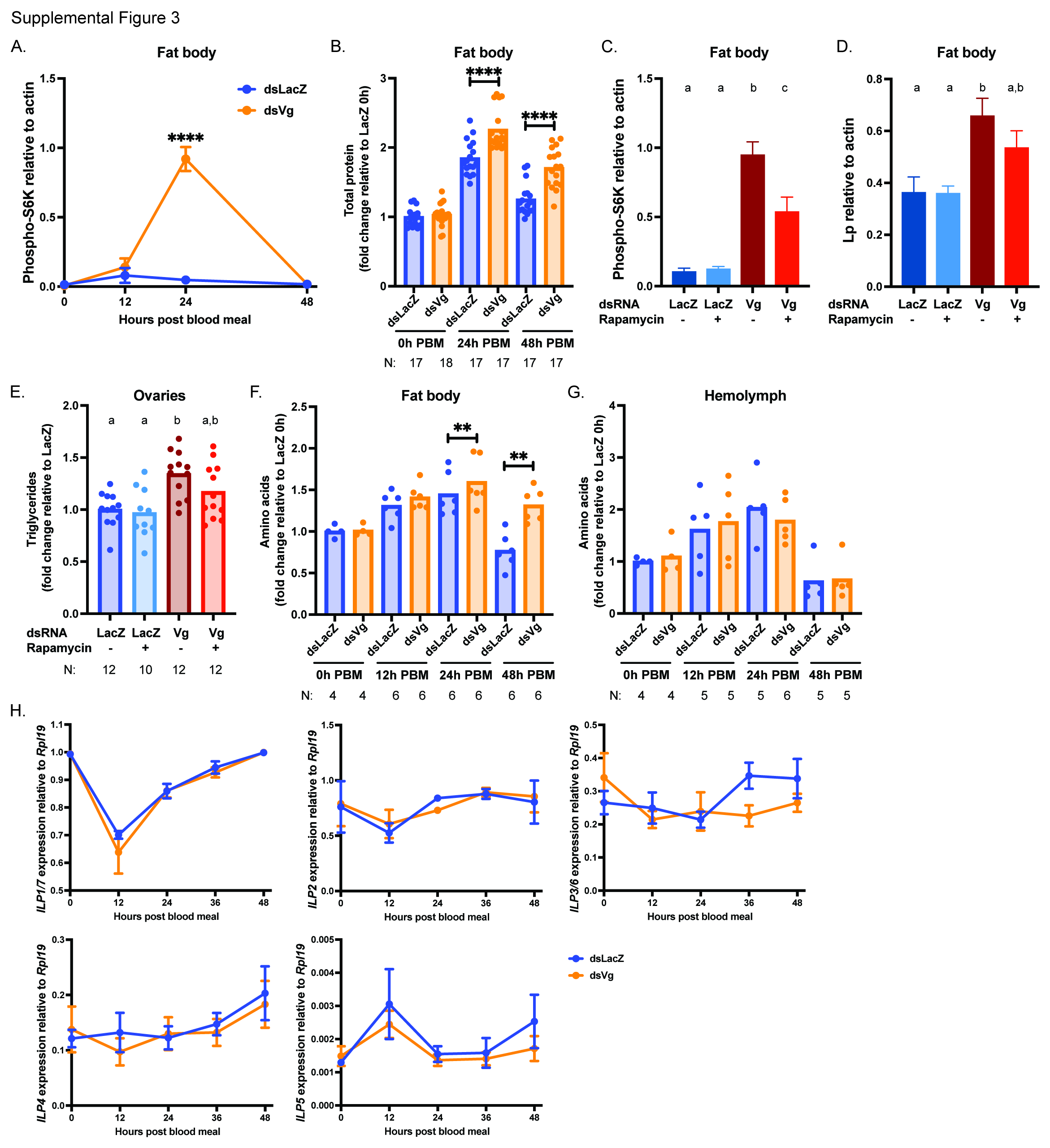

Supplement: S3 Fig — (A) Western blot quantification from Fig 3B showing an increase in phospho-S6K levels in the fat body upon Vg knockdown; samples of five tissues, three biological replicates (REML variance component analysis: **** = p < 0.0001). (B) Fold change in protein levels, measured by Bradford assay of the fat body are increased in dsVg females; each dot is representative of three fat bodies; N = number of samples of three tissues, pooled from three biological replicates (REML variance component analysis by timepoint: **** = p < 0.0001). (C) Western blot quantification from Fig 3D showing a decrease in phospho-S6K levels in the fat body upon rapamycin treatment; samples of five tissues, three biological replicates (ANOVA). (D) Western blot quantification from Fig 3E showing Lp protein levels upon Vg knockdown and rapamycin treatment; samples of five tissues, four biological replicates (ANOVA). (E) Triglyceride levels measured in dsLacZ and dsVg ovaries upon 0.5 μL of 40 μM rapamycin treatment at 72h PBM and normalized to mean dsLacZ levels in each replicate; each dot is representative of ovaries pooled from three females; N = number of samples of three tissues, pooled from two biological replicates (ANOVA). (F) Fold change in free amino acid levels in the fat bodies of dsLacZ and dsVg females before blood meal and at 12h, 24h and 48h PBM; each dot is representative of five ovaries; N = number of samples of five tissues, pooled from three biological replicates (REML variance component analysis by timepoint: ** = p < 0.01). (G) Fold change in free amino acid levels in the hemolymph of dsLacZ and dsVg females before blood meal and at 12h, 24h and 48h PBM; each dot is representative of hemolymph collected from five females; N = number of samples of five hemolymphs, pooled from three biological replicates (REML variance component analysis by timepoint). (H) RT-qPCR of ILP expression levels relative to Rpl19 in the heads of dsLacZ and dsVg females; samples of ten tissues, ILP1/7: t [file pgen.1011145.s003.tif]

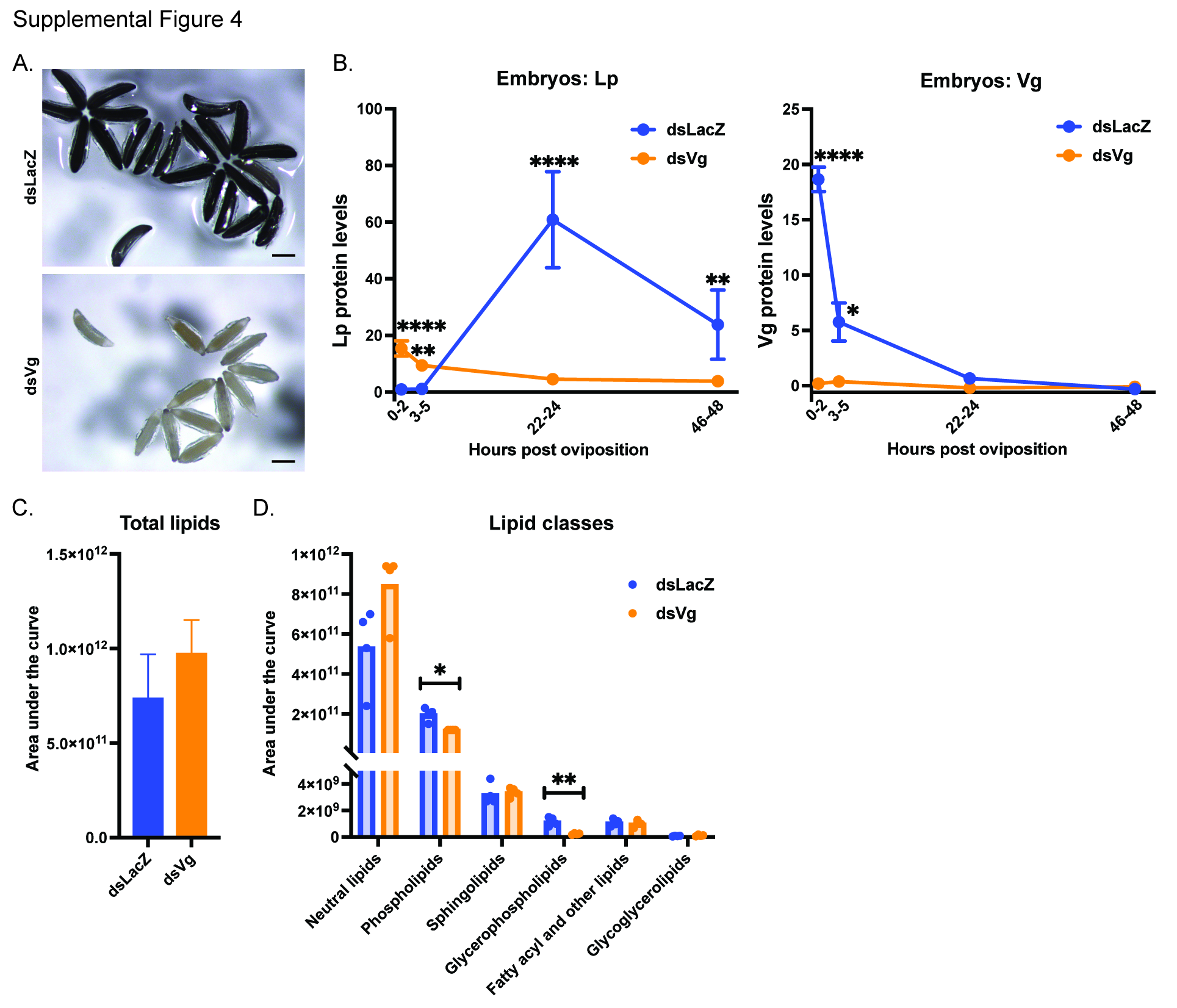

Supplement: S4 Fig — (A) Light microscopy of embryos from dsLacZ- and dsVg-derived females at 3–5h post oviposition; scale bar = 200 μm. (B) Western blot quantification of Lp and total Vg from Fig 4B; samples of 40 embryos, three biological replicates (REML variance component analysis: **** = p < 0.0001; ** = p < 0.01; * = p < 0.05). (C-D) Total lipids (C) and lipid classes (D) in dsLacZ- and dsVg-derived 200 embryos 3–5h post oviposition as determined by lipidomics, four biological replicates (Unpaired t tests, followed by FDR correction: * = p < 0.05; ** = p < 0.01). (TIF) [file pgen.1011145.s004.tif]

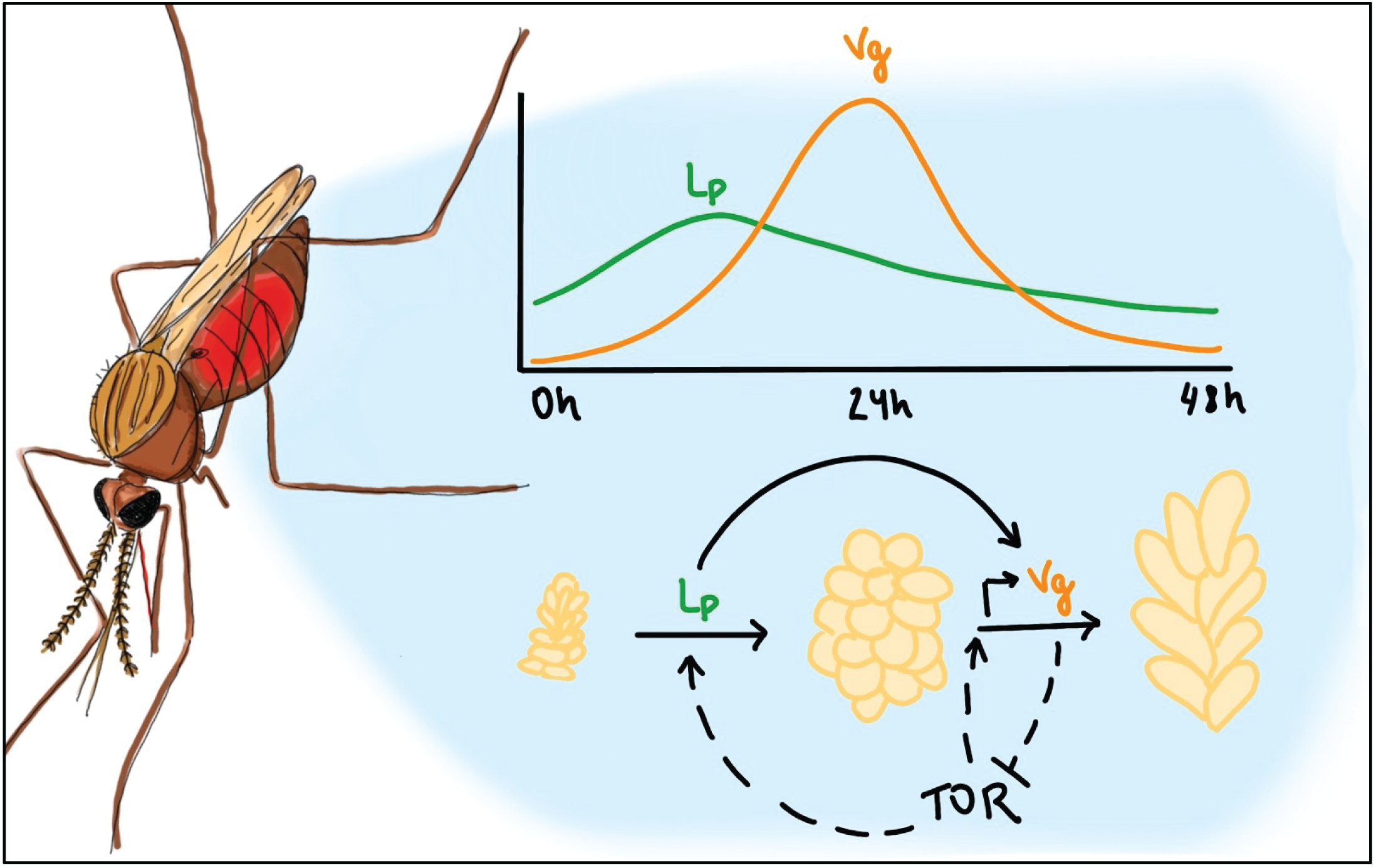

Supplement: S5 Fig — (TIF) [file pgen.1011145.s005.tif]
