# Supplementary material for: Precise coordination between nutrient transporters ensures fertility in the malaria mosquito Anopheles gambiae
Source: PLoS Genet. 2024 Jan 29;20(1):e1011145. doi: 10.1371/journal.pgen.1011145 (PMC10852252; doi:10.1371/journal.pgen.1011145)
Supplement: S1 Table — (DOCX) [file pgen.1011145.s006.docx]

**S1 Table.** **Vg amino acids and their decrease in embryos upon *Vg* depletion.**

| **Amino acid** | **Percent content in Vg** | **Decrease upon *Vg* KD in mothers (fold change)** | **Significance** |  |
| --- | --- | --- | --- | --- |
| Ser (S) | 8.50% | 2.2 | 0.09205 |  |
| Tyr (Y) | 8.00% | 52.6 | 0.00010 | *** |
| Phe (F) | 7.70% | 63.2 | 0.00000 | **** |
| Glu (E) | 6.50% | 1.2 | 0.33025 |  |
| Ala (A) | 6.30% | 2.0 | 0.02859 | * |
| Asp (D) | 6.30% | 2.1 | 0.15012 |  |
| Lys (K) | 6.20% | 10.0 | 0.00000 | **** |
| Gln (Q) | 6.10% | 1.1 | 0.88614 |  |
| Val (V) | 5.80% | 3.1 | 0.00005 | **** |
| Asn (N) | 5.60% | 5.5 | 0.00006 | **** |
| Leu (L) | 5.60% | 3.7 | 0.00024 | *** |
| Gly (G) | 4.40% | 2.2 | 0.02060 | * |
| Pro (P) | 4.40% | 1.4 | 0.01066 | * |
| Thr (T) | 4.40% | 3.9 | 0.00015 | *** |
| Arg (R) | 3.90% | 2.9 | 0.00012 | *** |
| Ile (I) | 3.20% | 8.7 | 0.00001 | **** |
| His (H) | 2.90% | 1.0 | 0.69815 |  |
| Met (M) | 2.20% | 4.9 | 0.00010 | *** |
| Cys (C) | 1.10% | Not detected |  |  |
| Trp (W) | 0.80% | 13.2 | 0.00002 | **** |
