# Supplementary material for: Precise coordination between nutrient transporters ensures fertility in the malaria mosquito Anopheles gambiae
Source: PLoS Genet. 2024 Jan 29;20(1):e1011145. doi: 10.1371/journal.pgen.1011145 (PMC10852252; doi:10.1371/journal.pgen.1011145)
Supplement: S4 Table — (DOCX) [file pgen.1011145.s009.docx]

**S4 Table. RT-qPCR primer sequences.**

| **Gene** | **Primer** | **Citation,  if previously published** |
| --- | --- | --- |
| *Rpl19* | F CCAACTCGCGACAAAACATTC  R ACCGGCTTCTTGATGATCAGA | [1] |
| *Lp* | F CAGCCAGGATGGTGAGCTTAA  R CACCAGCACCTTGGCGTT | [2] |
| *Vg* | F CCGACTACGACCAGGACTTC  R CTTCCGGCGTAGTAGACGAA | [2] |
| *ILP1/7* | F GCAAAAAGTCCGAGAATCTACTGATGA  R CGAACGATCGTTCAATGTGTGGA |  |
| *ILP2* | F CTACCTCTACGCCCAACAGC  R CGTGTACATAATCTGTGCGATAGTG |  |
| *ILP3/6* | F GGTAAAGGTACTGTCCTTCCTG  R AGTATCTGCTGCGTGTTGTC | [3] |
| *ILP4* | F TCTCCGAAAGAACACAGTTGA  R GGTTTCTGCCTGAACCACAT | [3] |
| *ILP5* | F GTGGCACCAGGAGAGTCATT  R GCCCAGTACAGATGGCGTAT |  |

**References**

1. Rogers DW, Whitten MM, Thailayil J, Soichot J, Levashina EA, Catteruccia F. Molecular and cellular components of the mating machinery in Anopheles gambiae females. Proc Natl Acad Sci U S A. 2008;105(49):19390-5. Epub 20081126. doi: 10.1073/pnas.0809723105. PubMed PMID: 19036921; PubMed Central PMCID: PMC2614771.

2. Baldini F, Gabrieli P, South A, Valim C, Mancini F, Catteruccia F. The interaction between a sexually transferred steroid hormone and a female protein regulates oogenesis in the malaria mosquito Anopheles gambiae. PLoS Biol. 2013;11(10):e1001695. Epub 20131029. doi: 10.1371/journal.pbio.1001695. PubMed PMID: 24204210; PubMed Central PMCID: PMC3812110.

3. Arsic D, Guerin PM. Nutrient content of diet affects the signaling activity of the insulin/target of rapamycin/p70 S6 kinase pathway in the African malaria mosquito Anopheles gambiae. J Insect Physiol. 2008;54(8):1226-35. doi: 10.1016/j.jinsphys.2008.06.003. PubMed PMID: 18634792.
