# Supplementary material for: Precise coordination between nutrient transporters ensures fertility in the malaria mosquito Anopheles gambiae
Source: PLoS Genet. 2024 Jan 29;20(1):e1011145. doi: 10.1371/journal.pgen.1011145 (PMC10852252; doi:10.1371/journal.pgen.1011145)
Supplement: S5 Table — For RT-qPCR, at least three independent biological replicates of a gene expression timecourse were analyzed, except for ILP1, where one replicate was excluded as an outlier. Effect test outputs are reported here. Multiple comparisons were calculated using pairwise Student’s t tests at each timepoint followed by FDR correction (see S6 Table). KD = knock down; rand = random effect; FDR = false discovery rate. (DOCX) [file pgen.1011145.s010.docx]

**S5 Table**. Details of statistical tests and outputs are summarized for each figure. For RT-qPCR, at least three independent biological replicates of a gene expression timecourse were analyzed, except for ILP1, where one replicate was excluded as an outlier. Effect test outputs are reported here. Multiple comparisons were calculated using pairwise Student’s t-tests at each timepoint followed by FDR correction (see **S6 Table**). KD = knock down; rand = random effect; FDR = false discovery rate.

| **Fig** | **Comparison** | **Statistical test** | **Effect Test Outputs** |
| --- | --- | --- | --- |
| **1A** | Egg number  after *Lp* KD | Mann-Whitney | p<0.0001 |
| **1B** | TAG levels after  *Lp* KD (Ovaries) | Ln(x+1.1) transformation;  Linear Mixed Model  at each timepoint | (0h) dsRNA p=0.7964  replicate[rand] p=0.3755  (24h) dsRNA p<0.0001  replicate[rand] p=0.3400  (48h) dsRNA p<0.0001  replicate[rand] p=0.0109 |
| **1B** | TAG levels after  *Lp* KD (Midgut) | Ln(x+1.1) transformation;  Linear Mixed Model  at each timepoint | (0h) dsRNA p=0.2984  replicate[rand] p=0.3317  (24h) dsRNA p<0.0001  replicate[rand] p=0.3573  (48h) dsRNA p<0.0001  replicate[rand] p=0.3228 |
| **1B** | TAG levels after  *Lp* KD (Fat body) | Ln(x+1.1) transformation;  Linear Mixed Model  at each timepoint | (0h) dsRNA p=0.6966  replicate[rand] p=0.3286  (24h) dsRNA p=0.0002  replicate[rand] p=0.3261  (48h) dsRNA p=0.0020  replicate[rand] p=0.3290 |
| **2A** | Egg number  after *Vg* KD | Mann-Whitney test | p<0.0001 |
| **2B** | Fertility  after *Vg* KD | Kruskal-Wallis test | ds*LacZ* vs ds*Vg*: p<0.0001  ds*LacZ* vs ds*Vg*#2:  p<0.0001  ds*Vg* vs ds*Vg*#2:  p>0.05 |
| **2E** | Triglycerides after *Vg* KD  (Ovaries 48h) | Unpaired t-test on transformed data | p=0.0222 |
| **2F** | *Lp* mRNA expression  after *Vg* KD | 4^th^ root transformation;  Linear Mixed Model  followed by 5 post-hoc  t-tests (**S6 Table**) | timepoint p<0.0001  dsRNA p<0.0001  dsRNA x timepoint p=0.0032  replicate[rand] p=0.3090 |
| **3A** | *Vg* mRNA upon rapamycin treatment | Unpaired t-test | p<0.0001 |
| **3C** | *Lp* mRNA levels upon rapamycin treatment | ANOVA | LacZ Control vs. LacZ Rapamycin  p=0.9956  LacZ Control vs. Vg Control  p=0.0002  LacZ Control vs. Vg Rapamycin  p=0.2500  LacZ Rapamycin vs. Vg Control  p=0.0003  LacZ Rapamycin vs. Vg Rapamycin  p=0.3469  Vg Control vs. Vg Rapamycin  p=0.0226 |
| **4D** | Triglycerides  after *Vg* KD  (Embryos) | Unpaired t-test | p=0.0496 |
| **S1A** | *Lp* mRNA expression  after *Lp* KD | 4^th^ root transformation;  Linear Mixed Model followed by 5 post-hoc  t-tests (**S6 Table**) | timepoint p<0.0001  dsRNA p<0.0001  dsRNA x timepoint p=0.0004  replicate[rand] p=0.478 |
| **S1B** | *Vg* mRNA expression  after *Lp* KD | 4^th^ root transformation;  Linear Mixed Model followed by 5 post-hoc t-tests (**S6 Table**) | timepoint p<0.0001  dsRNA p=0.044  dsRNA x timepoint p=0.0001  replicate[rand] p=0.536  replicate x timepoint[rand] p=0.033 |
| **S1C** | Vg protein expression  after *Lp* KD (Ovaries) | Ln(x+1) transformation;  Linear Mixed Model followed by 3 post-hoc t-tests (**S6 Table**) | timepoint p<0.0001  dsRNA p=0.0103  dsRNA x timepoint p=0.0192  replicate[rand] p=0.3836 |
| **S1C** | Vg protein expression  after *Lp* KD  (Fat body) | No transformation;  Generalized  Linear Model  followed by 3 post-hoc  t-tests (**S6 Table** | timepoint p<0.0001  dsRNA p=0.0148  dsRNA x timepoint p<0.0001  replicate p=0.0012 |
| **S1E** | Fertility  after *Lp* KD | Mann-Whitney test | p<0.0001 |
| **S1F** | Ecdysteroids  after *Lp* KD | Unpaired t-test | p>0.05 |
| **S2A** | *Vg* mRNA expression  after *Vg* KD | 8^th^ root transformation;  Linear Mixed Model followed by 5 post-hoc t-tests (**S6 Table**) | timepoint p<0.0001  dsRNA p<0.0001  dsRNA x timepoint p=0.0068  replicate[rand] p=0.6864 |
| **S2B** | Protein levels after *Vg* KD  (Ovaries) | No suitable transformation;  Generalized  Linear Model  at each timepoint | (0h) dsRNA p=0.8072  replicate p=0.4399  (24h) dsRNA p=0.0044  replicate p=0.0002  (48h) dsRNA p<0.0001  replicate p=0.0092 |
| **S2C** | Amino acid levels after *Vg* KD (Ovaries) | Square root transformation;  Linear Mixed Model  at each timepoint | (0h) dsRNA p=0.5067  replicate[rand] p=0.4232  (24h) dsRNA p=0.8833  replicate[rand] p=0.4843  (48h) dsRNA p=0.0163  replicate[rand] p=0.3562 |
| **S2D** | *Lp* mRNA expression after *Vg* KD#2 | 4^th^ root transformation;  Linear Mixed Model followed by 3 post-hoc  t-tests (**S6 Table**) | timepoint p=0.0004  dsRNA p=0.1804  dsRNA x timepoint p=0.0409  replicate[rand] p=0.9751 |
| **S2E** | Lp protein expression after  *Vg* KD (Ovaries) | Square root (x+0.03) transformation;  Linear Mixed Model followed by 3 post-hoc  t-tests (**S6 Table**) | timepoint p<0.0001  dsRNA p=0.0100  dsRNA x timepoint p=0.1130  replicate[rand] p=0.8073 |
| **S2E** | Lp protein expression after  *Vg* KD (Fat body) | No transformation;  Linear Mixed Model followed by 3 post-hoc t-tests (**S6 Table**) | timepoint p=0.0004  dsRNA p=0.0025  dsRNA x timepoint p=0.5277  replicate[rand] p=0.6241 |
| **S3A** | Protein levels  after *Vg* KD  (Fat body) | Cube root transformation;  Linear Mixed Model  at each timepoint | (0h) dsRNA p=0.4585  replicate[rand] p=0.3221  (24h) dsRNA p<0.0001  replicate[rand] p=0.3232  (48h) dsRNA p<0.0001  replicate[rand] p=0.3260 |
| **S3B** | pS6K expression  after *Vg* KD (Fat body) | No suitable transformation;  Generalized  Linear Model  followed by 4 post-hoc  t-tests (**S6 Table**) | timepoint p<0.0001  dsRNA p=0.1251  dsRNA x timepoint p<0.0001  replicate p=0.0084 |
| **S3C** | pS6K protein levels upon rapamycin treatment | ANOVA | LacZ Control vs. LacZ Rapamycin  p=0.9976  LacZ Control vs. Vg Control  p<0.0001  LacZ Control vs. Vg Rapamycin  p=0.0072  LacZ Rapamycin vs. Vg Control  p<0.0001  LacZ Rapamycin vs. Vg Rapamycin  p=0.0100  Vg Control vs. Vg Rapamycin  p=0.0102 |
| **S3D** | Lp protein levels upon rapamycin treatment | ANOVA | LacZ Control vs. LacZ Rapamycin  p>0.9999  LacZ Control vs. Vg Control  p=0.0185  LacZ Control vs. Vg Rapamycin  p=0.2184  LacZ Rapamycin vs. Vg Control  p=0.0172  LacZ Rapamycin vs. Vg Rapamycin  p=0.2044  Vg Control vs. Vg Rapamycin  p=0.4834 |
| **S3E** | Triglycerides  upon rapamycin treatment | ANOVA | LacZ Control vs. LacZ Rapamycin  P=0.9860  LacZ Control vs. Vg Control  p=0.0030  LacZ Control vs. Vg Rapamycin  p=0.2565  LacZ Rapamycin vs. Vg Control  p=0.0018  LacZ Rapamycin vs. Vg Rapamycin  p=0.1618  Vg Control vs. Vg Rapamycin  p=0.2566 |
| **S3F** | Amino acid levels after *Vg* KD  (Fat body) | No transformation  Linear Mixed Model  at each timepoint | (0h) dsRNA p=0.8672  replicate[rand] p=0.3832  (12h) dsRNA p=0.1087  replicate[rand] p=0.3257  (24h) dsRNA p=0.0064  replicate[rand] p=0.3201  (48h) dsRNA p=0.0027  replicate[rand] p=0.4067 |
| **S3G** | Amino acid levels after *Vg* KD (Hemolymph) | No transformation  Linear Mixed Model  at each timepoint | (0h) dsRNA p=0.7177  replicate[rand] p=0.7615  (12h) dsRNA p=0.6672  replicate[rand] p=0.4125  (24h) dsRNA p=0.2457  replicate[rand] p=0.3536  (48h) dsRNA p=0.8222  replicate[rand] p=0.7415 |
| **S3H** | *ILP1/7* mRNA expression  after *Vg* KD  (2 replicates) | arcsine transformation;  Linear Mixed Model  No post-hoc t-testing | timepoint p<0.0001  dsRNA p=0.426 |
|  | *ILP2* mRNA expression  after *Vg* KD | arcsine transformation;  Linear Mixed Model  No post-hoc t-testing | timepoint p=0.0089  dsRNA p=0.920 |
|  | *ILP3/6* mRNA expression  after *Vg* KD | No transformation;  Linear Mixed Model  No post-hoc t-testing | timepoint p=0.248  dsRNA p=0.367 |
|  | *ILP4* mRNA expression  after *Vg* KD | No transformation;  Linear Mixed Model  No post-hoc t-testing | timepoint p=0.002  dsRNA p=0.408 |
|  | *ILP5* mRNA expression  after *Vg* KD | 5^th^ root transformation;  Linear Mixed Model  No post-hoc t-testing | timepoint p=0.002  dsRNA p=0.241 |
| **S4B** | Lp protein  expression after *Vg* KD (Embryos) | Ln(x+1) transformation;  Linear Mixed Model followed by 4 post-hoc  t-tests (**S6 Table**) | timepoint p=0.0011  dsRNA p=0.9913  dsRNA x timepoint p<0.0001 replicate[rand] p=0.4256 |
| **S4B** | Vg protein  expression after *Vg* KD (Embryos) | Ln(x+0.7) transformation;  Linear Mixed Model followed by 4 post-hoc  t-tests (**S6 Table**) | timepoint p=0.0002  dsRNA p=0.0003  dsRNA x timepoint p=0.0005  replicate[rand] p=0.3706 |
| **S4C** | Embryo  total lipids | Unpaired t-test | p=0.1606 |
| **S4D** | Lipids  after *Vg* KD (Embryos) | Unpaired t-tests | **S6 Table** |
