# Supplementary material for: Precise coordination between nutrient transporters ensures fertility in the malaria mosquito Anopheles gambiae
Source: PLoS Genet. 2024 Jan 29;20(1):e1011145. doi: 10.1371/journal.pgen.1011145 (PMC10852252; doi:10.1371/journal.pgen.1011145)
Supplement: S6 Table — Tests were conducted for significant differences using an FDR of 0.05. See S5 Table. (DOCX) [file pgen.1011145.s011.docx]

**S6 Table. Details of post-hoc statistical testing.** Tests were conducted for significant differences using an FDR of 0.05. See **S5 Table**.

| **2F**  ***Lp* after *Vg* KD** | **p-value** | **FDR-adjusted  p-value** | **Significant?** |
| --- | --- | --- | --- |
| ds*LacZ* – ds*Vg* at 0h | 0.6414 | 0.6414 | No |
| ds*LacZ* – ds*Vg* at 12h | 0.0731 | 0.0914 | No |
| ds*LacZ* – ds*Vg* at 24h | 4.13 x 10^-6^ | 2.07 x 10^-5^ | Yes |
| ds*LacZ* – ds*Vg* at 36h | 0.0020 | 0.0050 | Yes |
| ds*LacZ* – ds*Vg* at 48h | 0.0450 | 0.0750 | No |
| **S1B**  ***Vg* after *Lp* KD** | **p-value** | **FDR-adjusted  p-value** | **Significant?** |
| ds*LacZ* – ds*Lp* at 0h | 0.9198 | 0.9198 | No |
| ds*LacZ* – ds*Lp* at 12h | 0.7704 | 0.9198 | No |
| ds*LacZ* – ds*Lp* at 24h | 0.0029 | 0.0048 | Yes |
| ds*LacZ* – ds*Lp* at 36h | 0.0028 | 0.0048 | Yes |
| ds*LacZ* – ds*Lp* at 48h | 7.55 x 10^-5^ | 3.78 x 10^-4^ | Yes |
| **S1A**  ***Lp* after *Lp* KD** | **p-value** | **FDR-adjusted  p-value** | **Significant?** |
| ds*LacZ* – ds*Lp* at 0h | 2.64 x 10^-5^ | 6.60 x 10^-5^ | Yes |
| ds*LacZ* – ds*Lp* at 12h | 4.36 x 10^-7^ | 2.18 x 10^-6^ | Yes |
| ds*LacZ* – ds*Lp* at 24h | 0.0138 | 0.0173 | Yes |
| ds*LacZ* – ds*Lp* at 36h | 0.8884 | 0.8884 | No |
| ds*LacZ* – ds*Lp* at 48h | 2.67 x 10^-4^ | 4.45 x 10^-4^ | Yes |
| **S1C (Ovaries)** | **p-value** | **FDR-adjusted p-value** | **Significant?** |
| ds*LacZ* – ds*Lp* at 0h | 0.7766 | 0.7766 | No |
| ds*LacZ* – ds*Lp* at 24h | 0.6119 | 0.7766 | No |
| ds*LacZ* – ds*Lp* at 48h | 0.0009 | 0.0027 | Yes |
| **S1C (Fat body)** | **p-value** | **FDR-adjusted p-value** | **Significant?** |
| ds*LacZ* – ds*Lp* at 0h | 0.7447 | 0.7447 | No |
| ds*LacZ* – ds*Lp* at 24h | 0.1780 | 0.2670 | No |
| ds*LacZ* – ds*Lp* at 48h | 2.86 x 10^-10^ | 8.56 x 10^-10^ | Yes |
| **S2A**  ***Vg* after *Vg* KD** | **p-value** | **FDR-adjusted  p-value** | **Significant?** |
| ds*LacZ* – ds*Vg* at 0h | 0.9000 | 0.9000 | No |
| ds*LacZ* – ds*Vg* at 12h | 0.0057 | 0.0115 | Yes |
| ds*LacZ* – ds*Vg* at 24h | 2.94 x 10^-5^ | 1.47 x 10^-4^ | Yes |
| ds*LacZ* – ds*Vg* at 36h | 0.0069 | 0.0115 | Yes |
| ds*LacZ* – ds*Vg* at 48h | 0.5501 | 0.6877 | No |
|  |  |  |  |
| **S2D**  ***Lp* after *Vg* KD#2** | **p-value** | **FDR-adjusted  p-value** | **Significant?** |
| ds*LacZ* – ds*Vg* at 0h | 0.3785 | 0.5678 | No |
| ds*LacZ* – ds*Vg* at 24h | 0.0098 | 0.0294 | Yes |
| ds*LacZ* – ds*Vg* at 48h | 0.8191 | 0.8191 | No |
| **S2E (Ovaries)** | **p-value** | **FDR-adjusted p-value** | **Significant?** |
| ds*LacZ* – ds*Vg* at 0h | 0.9392 | 0.9392 | No |
| ds*LacZ* – ds*Vg* at 24h | 0.0210 | 0.0315 | Yes |
| ds*LacZ* – ds*Vg* at 48h | 0.0177 | 0.0315 | No |
| **S2E (Fat body)** | **p-value** | **FDR-adjusted p-value** | **Significant?** |
| ds*LacZ* – ds*Vg* at 0h | 0.1927 | 0.1927 | No |
| ds*LacZ* – ds*Vg* at 24h | 0.0133 | 0.0399 | Yes |
| ds*LacZ* – ds*Vg* at 48h | 0.0298 | 0.0447 | Yes |
| **S3A (Fat body)** | **p-value** | **FDR-adjusted p-value** | **Significant?** |
| ds*LacZ* – ds*Vg* at 0h | 0.9058 | 0.9058 | No |
| ds*LacZ* – ds*Vg* at 12h | 0.1829 | 0.3658 | No |
| ds*LacZ* – ds*Vg* at 24h | 8.06 x 10^-25^ | 3.22 x 10^-24^ | Yes |
| ds*LacZ* – ds*Vg* at 48h | 0.6257 | 0.8343 | No |
| **S4B**  **Lp after *Vg* KD** | **p-value** | **FDR-adjusted  p-value** | **Significant?** |
| ds*LacZ* – ds*Vg* at 0–2h | 4.42 x 10^-5^ | 8.85 x 10^-5^ | Yes |
| ds*LacZ* – ds*Vg* at 3–5h | 0.0010 | 0.0013 | Yes |
| ds*LacZ* – ds*Vg*  at 22–24h | 1.70 x 10^-5^ | 6.82 x 10^-5^ | Yes |
| ds*LacZ* – ds*Vg*  at 46–48h | 0.0027 | 0.0027 | Yes |
| **S4B  Vg after *Vg* KD** | **p-value** | **FDR-adjusted  p-value** | **Significant?** |
| ds*LacZ* – ds*Vg* at 0–2h | 1.50 x 10^-5^ | 5.98 x 10^-5^ | Yes |
| ds*LacZ* – ds*Vg* at 3–5h | 0.0093 | 0.0186 | Yes |
| ds*LacZ* – ds*Vg*  at 22–24h | 0.0914 | 0.1219 | No |
| ds*LacZ* – ds*Vg*  at 46–48h | 0.1227 | 0.1227 | No |
| **S4D  Lipids after *Vg* KD** | **p-value** | **FDR-adjusted  p-value** | **Significant?** |
| ds*LacZ* – ds*Vg* (Neutral lipids) | 0.0624 | 0.1248 | No |
| ds*LacZ* – ds*Vg* (Phospholipids) | 0.0035 | 0.0105 | Yes |
| ds*LacZ* – ds*Vg* (Sphingolipids) | 0.7860 | 0.7860 | No |
| ds*LacZ* – ds*Vg* (Glycerophospholipids) | 0.0010 | 0.0060 | Yes |
| ds*LacZ* – ds*Vg* (Fatty acyl and other lipids) | 0.7144 | 0.7860 | No |
| ds*LacZ* – ds*Vg* (Glycoglycerolipids) | 0.2275 | 0.3413 | No |
