# Supplementary material for: Precise coordination between nutrient transporters ensures fertility in the malaria mosquito Anopheles gambiae
Source: PLoS Genet. 2024 Jan 29;20(1):e1011145. doi: 10.1371/journal.pgen.1011145 (PMC10852252; doi:10.1371/journal.pgen.1011145)
Supplement: Attachment — Submitted filename: Response to reviewers comments_FINAL.docx [file pgen.1011145.s013.docx]

**Reviewer #1:** The manuscript "Interplay between nutrient transporters ensures fertility in the malaria mosquito Anopheles gambiae" presented by Stryapunina and collaborators, examines the importance of Vg and Lp to ensure the reproduction of A. gambiae. The work is well done, with solid data, but some experiments or results have confusing descriptions of oocytes, egg chambers, eggs, or embryos, making it difficult to understand certain parts. Issues that can be improved by revising the manuscript. The authors propose to demonstrate the possible control that Vg and Lp exert on each other to control the reproduction of this mosquito species. To do this, molecular, microscopic, and analytical tools were used. Demonstrating at the end that the two proteins are necessary to complete the reproduction of A. gambie, as it happens with other insect species, but did not finish defining how the interaction between these two proteins occurs. With the work done using different methodologies, the authors can have more data and detail about what happens in the ovaries or fat body of the treated females, information that would improve the present manuscript. There are several details that I go on to describe below:

We thank Reviewer 1 for their valuable comments regarding our manuscript. We have answered their individual points below:

Material and Methods. Lines 476-477. The authors wrote: Virgin females were dissected 3-7 days after blood-feeding, and the egg clutches were counted. If females must be dissected (as indicated), these “eggs” are not still oviposited. Please, define at which stage an egg chamber is considered an egg.

Egg development is fully completed by 3 days post blood feeding in *An. gambiae* mosquitoes. We dissected virgins because we were assessing egg development at or after this stage (3 days) and virgin females do not oviposit their eggs. We have clarified this point in the manuscript (440-444). Referring to developed oocytes as eggs even if they are still within the ovaries is standard practice in published work in the field (Rono et al., PLOS Pathogens, 2010; Werling et al., Cell, 2019).

Must it have the chorion completed?

By 3 days post blood feeding the chorion is fully formed. However, it only melanizes post oviposition, unless the egg is unfertilized. (Chapter 19 - Oogenesis, p356, in The Biology of Mosquitoes, A. N. Clements, 1992)

At which stage of development are the egg chambers of the dissected treated females?

The eggs of both ds*Vg*-treated and control groups are at stage V in development (Chapter 19 - Oogenesis, p345, in The Biology of Mosquitoes, A. N. Clements, 1992).

Moreover, in the introduction (lines 59-60) is stated that the reproductive cycle culminates in the development of a full set of eggs in about 2-3 days. Why the dissections were done 3-7 days after blood feeding?

After the eggs are fully developed at 3 days post blood feeding, they do not further mature or change in number, as virgin females do not oviposit and we never observe resorption in laboratory conditions in the time frame of 3–7 days. To support this, please see our attached data below. In this experiment, eggs were dissected from ds*Vg* and ds*LacZ* females at 65 hours and 7 days post blood feeding. No difference is observed between the two timepoints (Kruskal-Wallis, p=0.0019, ** = p < 0.01).

Counting eggs at 3–7 days post blood feeding is done for convenience, and is standard procedure in the lab. We have added a sentence in the methods to clarify this point (440–444).

Lines 139-142. More than an atypical localization it seems that Vg is not completely released from the fat body.

The reviewer is correct and indeed we wrote that Vg persists in the fat body in line 141. In the revised manuscript, we have added that this might be due to impaired release of the protein by the fat body (now 158).

Is it also accumulated in the hemolymph?

We did not check Vg levels in the hemolymph as we only focussed on the tissue in which it is synthesized (fat body) and where it is targeted to (ovaries).

To allow the protein to reach the oocyte the VgR must be active. Is the VgR present in the oocyte membrane?

It has previously been shown in many insect species that VgR is present on the oocyte membrane (e.g. *A. aegypti*: Sappington 1995 PMID7633468; *D. melanogaster*: Schonbaum 1995 PMID7878005, 2000 PMID10679010). However, this has not been tested in *An. gambiae* and species-specific anti-VgR antibodies are not available, but we expect this to be the case.

At 24h PBM, the Vg levels in ovaries seem similar (Figure S1C) in dsLp and controls. However, in the image (Figure S1D) the ovaries are less developed. Do you have any hypothesis to explain that? Later, at 48 hours PBM the egg chambers appear completely reabsorbed.

The reviewer is correct in their observation. Indeed the ovaries are less developed as they have reduced lipid accumulation at 24h PBM, as shown in Fig 1B.

Line 146. Indicate Figure 1D after “ovaries”. In the next line indicates Figure 1C.

We thank the reviewer for pointing out these incorrect referrals to figures; We have since added “Fig 1D” (163) and “Fig 1C” (164).

Line 146. Figure 1D. From this figure, it seems that follicular cells were affected by Lp depletion. Are they in apoptosis? By the way, did you observe the nurse cells? Are they also affected? It is affected the LpR in oocytes?

The only visible microscopic difference was related to the size of the yolk bodies in the oocyte. We have performed the ApopTag assay in Lp-depleted ovaries and the results were inconclusive, so these data were not included in the manuscript. We did not analyze the nurse cells in the few eggs that developed, nor did we assess LpR expression, and cannot comment on whether they were affected.

Line 147. Vg does not disappear from western blots. It is not detected.

We have amended the wording to “decreased in intensity” (163).

Figure 1 legend. Regarding Fig. 1A, the authors say that "each dot represents eggs per female", and "N= number of females"(86 in the case of controls). However, the legend further refers to the statistics and goes saying "(Mann-Whitney: ****= p<0.0001; FOUR biological replicates)"(capitals are mine). What is finally the number of replicates? A similar confusion appears in panels B and C, and in diverse panels in Figure 2, Suppl. Figure 2 and Suppl. Figure 3.

We performed four biological replicates using 20–30 females per replicate. We have clarified this point by changing legends to “N= number of females, pooled from X biological replicates” in all figures. We have provided numbers of tissues in each sample and numbers of replicate samples in other panels, as appropriate. Full details of numbers of females used are provided in the methods section. We have also provided the supporting numerical data for each biological replicate in S7 Table.

Lines 180-182. After dsVg treatments, the depletion of Vg expression is complete (Fig S2 and Fig 2G). Can the authors explain why an important number of females can develop a similar number of “eggs” than control females? (Fig 2A).

Indeed this is an interesting point that we have also debated in our group. We conclude that the initial steps of oocyte development are controlled by lipid incorporation directed by Lp. By the time Vg is active, oocyte development has started and the resultant number of eggs is not impacted as severely in Vg knockdown, as upon Lp knockdown. However, the resulting eggs lack yolk, leading to a dramatic infertility phenotype. To clarify this point, in the manuscript we refer to these two phases both in the results and in the discussion (341 onwards).

Indicate in the legend if these “eggs” are quantified from ovaries of 3-7 day PBM females, as indicated in material and methods.

Legends from Fig 1A and Fig 2A were amended to reflect this information.

By the way, the interval of 3-7 days is too large in a mosquito life to describe phenotypes, perhaps the authors can provide the data corresponding to each day and the result could show a kinetic of egg chamber development after dsVg treatment.

We have addressed this comment previously. Egg numbers do not change in this timeframe in virgin females.

Lines 178- 188. From the title of the section and the title of Figure 2 legend, a description of oocyte development (or egg chamber development) was expected. However, in the text and Figure, all the data refer to eggs. The explanation in material and methods (see the former comment) indicates that females were dissected to quantify the number of eggs (perhaps egg chambers). In line 181, the authors noted that a decrease in Vg led to a significant reduction in egg count. Should we assume that Figure 2A depicts oviposited eggs and Figure 2B portrays the larvae hatching from those eggs? Please, clarify these points. Indicate if these females are virgins or fecunded.

In our original submission, we have used the term oocyte to signify eggs while they were still developing within the female, and eggs to refer to fully developed follicles from 3 days PBM, even if they are not laid. Thus we have used both oocyte and egg terms. In this revised submission we have now been more careful in our usage to prevent confusion, referring throughout more specifically to eggs or oocytes. To address the latter points made by the reviewer, Fig 2A depicts the number of eggs in ovaries dissected per virgin female at 3–7 days PBM (which has now been added to the legend, see above comment), while Fig 2B depicts the larval hatch rate from eggs laid by mated females in a separate experiment. We have also clarified that Fig 2C and 2D refer to developing oocytes.

Line 183. The authors wrote: “no embryos hatching from Vg-depleted mothers” Which is the meaning of hatching in this sentence. Are you meaning hatching larvae from eggs?

We have changed the text to reflect this comment: “no larvae hatching from eggs oviposited by mated Vg-depleted mothers (182)”.

Line 190. From Figure 2D, it seems that the nurse cells are bigger 24 hours after the dsVg treatment than in control females. Any hypothesis explaining that?

While there may be a small delay in development in the oocytes in the Vg-depleted group as reported in Fig 2D, this was not reproducibly observed.

Line 195. Change silencing by depleting. Revise the document, the mRNA cannot be silenced by dsRNA.

We have changed “silenced” to “depleted” throughout the manuscript.

Lines 195-200. After the peak at 24 hours, Lp expression reaches the control levels at 48 h PBM. Do you have any suggestions for this return to normal levels?

The effects on Lp are maximal at the time of Vg expression, which peaks at 24h PBM. We have added this explanation (215–216).

Line 237. Treatment with rapamycin has resulted in a 50% decrease in Vg. Have higher doses been attempted to further decrease Vg?

We did not attempt higher (or lower) doses as the dose we used was already double the amount shown to be effective (by injection and feeding) in a recently published work (Feng et al., PNAS, 2021, PMID: 33626094).

In dsVg-treated females, there is an increase in p-6SK. Do you analyze the p-6SK in dsLp-treated females? The changes in TOR signaling only happen when Vg is depleted?

We did not analyze p-S6K upon Lp depletion so we cannot conclude whether TOR signaling is impacted upon Lp KD. In future studies it would be interesting to also study TOR signaling after Lp depletion.

Line 241. Change “post PBM” by “PBM”

We have changed “post PBM” to “PBM” (230).

Line 243. Add (Figure 3B) after “controls”

We have added a reference to Fig 3B and S3A Fig (232).

Lines 312-327. Please revise the description in this section. It is confusing the use of “embryos” when the authors are making references to eggs. For example:
Line 314. Authors say “the mechanisms causing infertility in embryos”. Please revise, the word infertility is not correct in this sentence. Perhaps arrest of development, or egg infertility.

We have changed “infertility” to “lethality” (281).

Line 315. Embryos cannot be fertilized. The eggs can be fertilized.

We have changed “embryos” to “eggs” (282).

Line 322. Embryos are not deposited in mosquitos

We have changed “embryos deposited by Vg-depleted mothers” to “embryos from Vg-depleted mothers” (305).

Line. 356. Authors wrote: “(B) Lp is deposited into dsVg-derived embryos”. Lp was deposited in the eggs.

We have changed “embryos” to “oocytes” since this happens in the developing eggs (295).

Figure 4. Please explain the figures in panel A. What are showing the two images of controls? It seems that both are at the surface of the eggs. Considered to change one of them for an image at a higher magnification that allows the comparison with the image from the treated embryo.

We have modified the figure to enlarge the panels and add a zoomed-in panel of the control embryo. We have also improved clarity by changing the color palette to grayscale as requested by another reviewer. The explanation of the figure is given in lines 280-288 of the results section. The control embryos are in the cellular blastocyst stage, which is characteristic of normally developing embryos at 3–5h post oviposition. In this stage, the nuclei are highly organized at the edge of the embryo, underneath the chorion. However, in Vg-depleted embryos the nuclei have divided but are disorganized and have not yet finished their migration towards the chorion.

Line 586. It is indicated “5 2mm glass” probably it must say 5.2 mm

The original wording is correct but we agree it was misleading and have therefore changed it to “five 2 mm glass beads” for clarity (562–563).

Through the document change ml by mL. The same, change μl by μL and nl by nL
Revise the correct use of “silence” after treatment with dsRNA. The RNAi do not silence the gene expression.

We have made these suggested changes throughout the manuscript.

**Reviewer #2:** This article reveals that LP and Vg are reciprocally regulated in Anopheles gambiae, functioning as two nutrient transporters that maintain the normal development of ovaries and ensure fertility. Silencing LP disrupts lipid transport, leading to the mis-regulation of Vg expression in follicles and enlargement of yolk granules, ultimately resulting in the cessation of egg production. Conversely, silencing Vg upregulates the expression of LP in the fat body, causing excessive lipid accumulation in the follicle and rendering females completely infertile. The regulation of LP by Vg is partially mediated by TOR signals. Additionally, the study found that Vg can control amino acid levels and protein synthesis by transporting amino acids, which may influence embryonic development. In brief, the manuscript's data is detailed, and the results appear reliable, but there are some issues that need to be addressed.
We thank Dr. Zhen Zou for signing his review and for his valuable comments on our manuscript.

Suggestions:
Major:
1. Figure 1, add the result of hatchability in Lp-silenced females.

Thank you for this suggested experiment. Following this suggestion, we have now performed this experiment in *Lp*-knockdown females. The vast majority (68 of 71) of mated blood fed *Lp*-knockdown females did not lay any eggs, precluding a strong assessment of fertility. Nevertheless, in those that laid, we observed a complete infertility phenotype as in *Vg* knockdown. We have added these data to the manuscript in S1 Fig.

2. Figure 1B, the authors measured the levels of triglycerides in the midgut and ovary, indicating triglycerides accumulate in the midgut and do not transfer from the midgut to the ovary. But why not test the content of triglycerides in fat body.

Thank you for this suggested experiment. We were primarily focused on examining levels of triglycerides in the midgut (a source of triglycerides following a blood meal) and the target tissue (ovaries). Nevertheless, we have determined levels of triglycerides in the fat body and see an accumulation after a blood meal when Lp is depleted. We have added these data to Fig 1B in the manuscript.

3. Supplemental Figure 1B (where Vg expression was measured only in fat bodies), whereas in supplemental Figure 1C fat bodies and ovaries were used. It is not comparable.

Thank you for this comment. We have focused on particular tissues in different Supplemental Figures because Vg is expressed specifically in the fat body of the mosquito. Thus, the mRNA levels are measured only in the fat body. Once it is transcribed, translated and folded in the fat body, it is then released into the hemolymph and travels to other tissues. The protein levels are thus measured in the tissues that either express Vg (fat body) or in which Vg accumulates (ovaries). We have clarified this background information in the introduction (67-70) and results (151-152) sections to explain our reasoning.

4. For Figure 2, Supplemental Figure 1 and 2, the control is actin sometimes and changes to Rpl19 in other cases.

We have used different controls depending on the type of experiment we are conducting. We use actin as the control when we measure protein levels in Western blots while we use Rpl19 as the control when we measure mRNA levels via RT-qPCR.

5. Figure 4B, the disappearance of actin, even if protein synthesis is inhibited, requires explanation, as actin, a housekeeping gene, must be present in living cells. Please provide a reasonable justification for this observation.

We would argue that actin does not disappear. Rather, at these early stages of embryonic development, it is at a concentration below the level of detection by this antibody on a Western blot. As the control embryo undergoes development it produces more actin, which can be visualized in later timepoints in Fig 4B. ds*Vg* embryos, however, die very early in development, and no longer have ‘living cells’. Thus, they never produce enough actin for it to be detected on the Western blot.

6. Problems with lipid metabolism. In Figure 2G, Lp protein increased in both fat body and ovary of Vg-silenced females. Meanwhile, in Figure 2E, triglyceride levels were also elevated in the ovary of Vg-silenced females. However, in Figure 4B, Lp protein decreased in the embryos of Vg-silenced females and in Figure 4D, and triglyceride levels were still elevated. How to explain this contradictory result?

We do not consider these results to be contradictory. In the embryos from Vg-depleted mothers (Fig 4B), Lp is deposited via inappropriate maternal deposition, leading to accumulation of triglycerides, and then does not change or maybe degrades slowly over time after the early death of the embryo at 0–2 h post oviposition. In control embryos, there is no maternal deposition of Lp, but *Lp* expression is instead induced at later time points after oviposition (Lp is visible in the blot of Fig 4B from 22–24h post-oviposition but not at the earlier stages immediately after oviposition). Elevated triglycerides levels at 3–5h post oviposition (Fig 4D) in embryos from Vg-depleted are due to elevated levels in the eggs (Fig 2E). We have now clarified these results in the text, adding additional information (305–312).

Importantly, in Figure 4D, supplemental 4B and 4C, no differences in total lipids were observed in dsVg-derived embryos relative to controls, but triglyceride levels increased, and some lipid levels decreased. These results only prove that lipids changed in Vg-silenced females, but the relationship between lipid alterations and reproduction is not explained, so it is recommended that further experimental validation or discussion of the lipid metabolism results is needed.

We have clarified our discussion of the lipid data. The increase in triglycerides in embryos in Fig 4D is caused by the earlier increased deposition of triglycerides in Vg-depleted ovaries. The total lipids do not show a significant increase because they include all lipids present in the embryo. However, the visible trend in total lipids is driven by the significant increase in triglycerides, a neutral lipid. In our discussion, our hypothesis is that embryonic lethality is caused by a lack of Vg-derived amino acids. Dr Zou is correct that we did not include the lipid observations as a potential cause of lethality in the discussion and we have since added a sentence to the discussion to reflect this possibility (372).

Minor:
7. Please ensure the consistent use of units and correct punctuation throughout the article. For instance, there are inconsistencies in punctuation, such as line 147: “2mm” and line 283: “40 μM”.

We have ensured our use of units is consistent throughout the manuscript. We have added a space between numbers and units where possible, with the exception of 1) the figures to save space, and 2) hours (h), which are very numerous.

8. Line 127-129, what does “without characterizing the phenotype” mean here?

Experiments to analyze ovarian development over time or female fertility were not conducted in these publications. We have modified our sentence to be more specific about previous work: “these studies did not characterize oocyte development over time and did not assess effects on fertility” (130).

9. Line 145-148, Whether the sample is ovaries (figure legend, line162) or eggs (result, line145).

We have amended this error. The sample is ovaries, and we have changed “eggs” to “ovarian follicles” (162).

10. Line 147, the phrase “largely disappeared” may not be the most accurate description of Figure 1C. Consider using more precise description.

We agree and have changed the description to “decreased in intensity in Western blots” (163).

11. Line 390, “Vg localization is affected (Figure 1C)” change to “Vg localization and accumulation is affected (Figure 1C)”.

We have changed the sentence as suggested (346).

12. In Figure 4B, it would be beneficial to include a biological statistical analysis to more intuitively illustrate the changes in yolk granules.

We have included a quantification of the data in S4 Fig.

13. In Supplemental Figure 3E, I'm curious why qPCR analysis of LP and Vg expression levels was performed solely on fat bodies and not on ovaries. Please clarify.

Both *Vg* and *Lp* are transcribed specifically in the fat body of the mosquito and there is no expression in the ovaries. We have specified this throughout the text.

**Reviewer #3:** In this paper, Stryapunina et al. describe the interplay between nutrient transporters Lipophorin (Lp) and Vitellogenin (Vg) to ensure fertility in the malaria mosquito Anopheles gambiae. The authors used a plethora of methods to describe the mutual relationship of studied factors and show effects on female mosquito physiology. The manuscript is well written, experiments are robust and well described and the story is easy to follow. Although the authors do not provide direct experimental evidence of how at a molecular level Lp and Vg mutually affect their expression and function, the work is on a track to uncover basic regulation of the gonotrophic cycle in a mosquito species, which is a deadly threat to many human populations, and thus, studies of its reproduction are of the highest interest. I have several major and minor comments on the manuscript.
We thank Reviewer 3 for their positive comments and excellent review of our manuscript.

Major comments
My major concern is the described relationship of Lp and Vg. Although the authors describe Lp and Vg relationship as an ‘interplay’ in the article title, there is no experimental proof, in the sense of a molecular mechanism, of a direct effect of Lp on Vg expression and Vg on Lp. Several intermediate steps between the Lp silencing and changed Vg levels, or Vg depletion and changed Lp levels could be included and drive the ‘interplay’. The authors themselves mention in the discussion that Lp expression induction upon Vg RNAi can be just a consequence of the presence of free amino acids, which were not incorporated in Vg but remained in the FB, activating the TOR signaling pathway. I need to admit that the authors are aware of what was just said and are mostly cautious about their conclusions, which is appropriate given the data they present.
This is a good point. We have endeavored not to overstate our results but fairly report our findings relating to the interactions between Lp, Vg and TOR, which may be indirect.

1) The authors mention in the introduction, that Lp shuttles not only neutral lipids from the midgut to the ovaries but also cholesterol. It is difficult to rule out the role of ecdysone from Lp/Vg interplay with Lp as a shuttle of ecdysone precursor cholesterol and a (putative) target of the ecdysone signaling pathway. Moreover, with ecdysone importance for Vg expression. In the Suppl. Fig 1B, Vg mRNA expression in the FB at 24h PBM is higher in control dsLacZ females compared to dsLp females. Could Lp-deprived females suffer from lower ecdysone production? Could that be reflected in the lower Vg transcription?

This was also our hypothesis, but we were surprised to see no difference in 20E levels around its peak at 26h PBM upon Lp knockdown. We have now added these data to S1 Fig. A possible explanation for this observation is that, after the first blood meal, ovary-synthesized E is produced from larval-derived cholesterol that is stored in ovaries and does not require Lp for transport. We have added this explanation to the text (164-171).

Further, Lp seems to be repressed by ecdysone signaling in An. gambiae (Werling et al. 2019) and Lp mRNA expression peaks at 12h PBM (Fig 2F; Werling et al., 2019), ahead of the ecdysone peak (Redfern 1982; Pondeville et al., 2008). However, the maximal Lp expression in the Vg-RNAi background is shifted to 24h PBM (when ecdysone and free amino acids are present) but drops at 36h and 48h PBM, when free amino acids are present in the FB (Suppl Fig 3), but ecdysone is dropping (mainly at 48h PBM). Could ecdysone (with amino acids?) function as a feedback loop to repress Lp expression (and function) and really coordinate Lp/Vg interplay? Do TOR and 20E signaling really exert opposite effects on Lp expression as suggested in the discussion? I believe those are serious questions, that should be considered, discussed, and hopefully supported by data.

Reviewer 3 makes an excellent point, and we now realize that our discussion of 20E, Vg and Lp needs to be more nuanced and to incorporate time. It is highly possible that 20E signaling keeps Lp levels low as observed at later time points (48h PBM). However, we believe the result observed by Werling *et al*. at 24h [increased Lp expression in dsEcR] is mediated by increased TOR signaling/reduced Vg expression, while additional EcR-controlled mediators, or EcR itself, are responsible for the decreased Lp expression at later time points.

Overall, we still believe that 20E and TOR signaling do indeed have opposite effects on *Lp* expression, since a repression of 20E signaling results in a sustained increase in Lp levels (Werling *et al*.), while when we suppress increased TOR signaling with rapamycin we see a decrease in the dsVg-mediated increase in Lp.

We have modified our discussion to introduce these points (386-398).

2) Vg protein level in the FB is higher in Lp-silenced females at 24h PBM. Does Lp directly block Vg release to the hemolymph? Or are ovaries already degenerating and thus not accumulating Vg even at 24h PBM and the excess of the Vg is just retained in the FB? The authors might discuss it a little more.

Reviewer 1 made a similar comment and we agree, and have added that there may be impaired release of Vg by the fat body (157).

3) The authors consistently mention the number of biological replicates, but the number of plotted and statistically analyzed samples/animals or FB/ovary pools – the total number ‘N’ – is sometimes missing or can be confused with the number of biological replicates. The authors should add ‘N’ to the figure: Fig 1B, 2E, Suppl Fig 2B-C etc.
Reviewer 1 made a similar comment. We have now clarified how many mosquitoes/samples were included and analyzed in each experiment. We have also added ‘N’ to the specified figures.

4) Gene symbols in many cases describing samples/animals upon dsRNA injection are often not in italics, especially dsLp and dsVg.

We apologize for any errors in italicisation. We have corrected these errors.

Minor comments:

Line 33-34 ‘Embryos deposited by Vg-depleted mothers are completely infertile…’ I think only Vg-depleted mothers are completely infertile.

We have changed “infertile” to “inviable” (33).

Line 217 (Suppl. Fig 1D) Please remove 12h and 36h time points (x-axis), as no values are plotted for those time points.

We have removed 12h and 36h from the x-axis.

Line 227 ‘Vg knockdown by second target …’ It is Vg, which is targeted by the second Vg RNAi fragment.

We have changed the sentence to ‘*Vg* knockdown by a second Vg RNAi fragment also results…’ (845)

Line 257 ‘that in normal conditions Vg expression represses TOR signaling’ – Is it really repression as we commonly understand it or does Vg expression lead to repressed TOR pathway?

We have changed the sentence to ‘Vg expression leads to repressed TOR signaling…’ (261)

Line 281 The Vg-knockdown mediated.

We have amended it to ‘Vg-knockdown mediated upregulation…’(242)

Line 286-287 ‘in the same four groups’ might suggest that the same samples as in Fig 3D were used, which was not the case. Please reword the caption accordingly.

We have changed the legend to ‘Western blot showing fat body Lp levels are increased in Vg-knockdown females at 48 h PBM, but this increase is repressed by rapamycin treatment.’ (248-250)

Line 350 (Figure 4A) It is difficult to see details in Figure 4A. Although the figure quality is supposed to be higher in the final version, the authors could change the blue channel color to white.

We have increased the size and quality of the images, and provided another zoomed-in inset to better visualize the control embryos. We have changed the color palette to grayscale for clarity. Please review the higher quality .tif files provided in this revision from the editorial management system.

Line 388 Is 20E ‘synthesized in the fat body’, or is it converted from ecdysone synthetized in ovaries?

We have changed the sentence to ‘During this early phase, as E is converted to 20E in the fat body, this…’ (343)

Line 424 Is it really repression as we commonly understand it? Or Vg expression leads to TOR signaling suppression.

We have changed the sentence to read ‘Vg expression leads to suppressed TOR signaling…’ (383)

Line 471 I am quite skeptical that the authors had dsRNA of 10 ug/uL concentration, especially if NanoDrop was used for quantification. NanoDrop measures the concentration linearly up to ~ 5 ug/uL and the higher concentration is just a linear approximation, not an accurate measurement. How confident the authors are in dsRNA concentration determination?

We quantified the concentration of dsRNA produced by *in vitro* transcription by diluting a small aliquot one in ten, yielding a concentration below 5 µg/µl that can be accurately measured by NanoDrop.

Line 476-477 Were females really dissected to do the egg counting?

Yes. Since virgin females were used for egg counts, they had not laid their clutches so they were dissected.

Line 489-490 Why were random primers used for the cDNA synthesis? Were they more reliable than oligo(dT) primers?

Using random primers for cDNA synthesis is a well-established method that provides more even coverage of the mRNA than oligo(dT) primers, which produce cDNA biased towards the 3’ end of the mRNA. Our qRT primers are not specifically designed to be positioned near the 3’ end of the mRNA and so we use random primers. They have been successfully used in previous publications e.g. Werling *et al.*, 2019.

Line 495 and Suppl. Table 4 Rpl19 and genes in the table in italics.

We have changed gene names to be in italics

Line 513 What does 25% bleach mean? Please provide the final concentration of the active substance(s).

We apologize for the error. We have changed the sentence to “25% household bleach (2% sodium hypochlorite final concentration) in 1x PBS”. (483)

Line 537-540 Was acetone part of the solution? Wasn’t rapamycin dissolved in DMSO only?

Rapamycin was dissolved in DMSO at 10mM. Acetone was then used as a volatile carrier solvent of this rapamycin/DMSO solution with the final concentrations being 40µM rapamycin and 2.4% DMSO (v/v) in acetone to perform topical applications of the compound to the insect cuticle. Use of acetone ensures even compound delivery and aids compound penetration of the cuticle and then rapidly evaporates. We have clarified this in the methods (508-513)

**Have all data underlying the figures and results presented in the manuscript been provided?**
Large-scale datasets should be made available via a public repository as described in the *PLOS Genetics* [data availability policy](https://urldefense.proofpoint.com/v2/url?u=http-3A__journals.plos.org_plosgenetics_s_data-2Davailability&d=DwMGaQ&c=WO-RGvefibhHBZq3fL85hQ&r=K-p4XIh6SN22fAwMAAk4PngbaNhbMPbjWIgWvpUHDRU&m=69adu8u-MZjJGjl3rhWuFVa8wQI-_yp05o5H2UQ-JlyTxrbZ7zvd0HQUHGqQmPzg&s=bUOpGXCwJOFE-ZX4PnflJqCvkOjbk2Jla1YLBhhGOX4&e=), and numerical data that underlies graphs or summary statistics should be provided in spreadsheet form as supporting information.

Reviewer #1: Yes

Reviewer #2: Yes

Reviewer #3: **No:** The authors should provide numerical data that underlies graphs in spreadsheet form as supporting information.

We have provided the numerical data in the supporting information (S7 Table) and in the Harvard Dataverse repository with identifier doi:10.7910/DVN/C5UOZS.

PLOS authors have the option to publish the peer review history of their article ([what does this mean?](https://urldefense.proofpoint.com/v2/url?u=https-3A__journals.plos.org_plosgenetics_s_editorial-2Dand-2Dpeer-2Dreview-2Dprocess-23loc-2Dpeer-2Dreview-2Dhistory&d=DwMGaQ&c=WO-RGvefibhHBZq3fL85hQ&r=K-p4XIh6SN22fAwMAAk4PngbaNhbMPbjWIgWvpUHDRU&m=69adu8u-MZjJGjl3rhWuFVa8wQI-_yp05o5H2UQ-JlyTxrbZ7zvd0HQUHGqQmPzg&s=J21xsxgBx5vpWiVwsHp_AJCY9Q9YXchoqIAxH7XSTUk&e=)). If published, this will include your full peer review and any attached files.


If you choose “no”, your identity will remain anonymous but your review may still be made public.


**Do you want your identity to be public for this peer review?** For information about this choice, including consent withdrawal, please see our [Privacy Policy](https://urldefense.proofpoint.com/v2/url?u=https-3A__www.plos.org_privacy-2Dpolicy&d=DwMGaQ&c=WO-RGvefibhHBZq3fL85hQ&r=K-p4XIh6SN22fAwMAAk4PngbaNhbMPbjWIgWvpUHDRU&m=69adu8u-MZjJGjl3rhWuFVa8wQI-_yp05o5H2UQ-JlyTxrbZ7zvd0HQUHGqQmPzg&s=3nTyu8ouIEqcMCXSwHibR5xPOOWRLWzlPjw4jFEm9Zg&e=).

Reviewer #1: No

Reviewer #2: **Yes:** Zhen Zou

Reviewer #3: No

We thank all reviewers for their comments and Dr. Zhen Zou for signing his review.
