# Supplementary material for: Precise coordination between nutrient transporters ensures fertility in the malaria mosquito Anopheles gambiae
Source: PLoS Genet. 2024 Jan 29;20(1):e1011145. doi: 10.1371/journal.pgen.1011145 (PMC10852252; doi:10.1371/journal.pgen.1011145)
Supplement: Attachment — Submitted filename: Response to Reviewer comments.docx [file pgen.1011145.s014.docx]

Reviewer's Responses to Questions

**Comments to the Authors:
Please note here if the review is uploaded as an attachment.**

Associate Editor: Thank you for revising the manuscript. The manuscript is almost ready for publication, but as you will see below, Reviewer #3 has some minor comments. I'd like to ask you to address these comments in the final round of review.

Reviewer #1: My comments have been addressed, and the manuscript fulfills my expectations.

We thank the reviewer for their comments and are very glad that we have sufficiently addressed their concerns.

Reviewer #2: I support the publication of this manuscript.

We are appreciative of the reviewer’s comments and their support of this publication.

Reviewer #3: In the manuscript by Stryapunina et al., the authors present a revised version of the original submission. I appreciate the authors for their response to the suggestions from all the reviewers and for providing new data. The changes incorporated into the manuscript substantially improved its quality, accuracy, and readability. The manuscript is ready to be accepted after dealing with only several small issues.

We thank the reviewer for their comments, and we agree that the changes have improved the final manuscript significantly.

I still think the authors should reconsider changing the title to reflect more the fact that the Lp/Vg interaction is most likely indirect, and the term interplay is not fully correct, from my point of view, interplay suggests either a physical interaction or direct involvement in the other’s protein actions. I understand that the term ‘interplay’ is “catchier”, but I would suggest something like (examples) Orchestration of nutrient transporters ensures fertility in the malaria mosquito Anopheles gambiae OR Precise coordination between nutrient transporters ensures fertility in the malaria mosquito Anopheles gambiae.

We have amended our title to avoid the potential misconception of a direct interaction. It is now “Precise coordination between nutrient transporters ensures fertility in the malaria mosquito Anopheles gambiae.”

The fact that the cholesterol from the larval stage seems to be used for ecdysteroidogenesis is interesting. It might safeguard the vitellogenic females from being dependent on blood meal-originated cholesterol and enable them to promptly start ecdysteroidogenesis and the first gonadotrophic cycle, without losing time for Lp to transport cholesterol from the gut.

This is our hypothesis, and we agree that this would potentially provide the vitellogenic female with an immediate source of cholesterol. It would be interesting to examine how 20E levels change following a second blood meal is ds*Lp* females, whose fat body stores of cholesterol would be expected to be depleted.

Lines 118-119 This is tightly linked to what I have already mentioned above. Do the authors expect the Vg to be the molecule, which is directly responsible for the termination of the Lp-mediated accumulation of lipids in the ovaries? Or is Vg a proxy of lacking amino acids?

We consider that it is the latter. We have changed the wording to “Lp is needed for the correct incorporation of Vg in the developing oocytes while Vg’s utilization of amino acids is required to terminate the Lp-mediated accumulation of lipids in the ovaries” (line 117).

Lines 152 might suggest the Vg mRNA is transcribed in the FB and Vg mRNA is incorporated into the developing oocyte. The authors could change it to (example) ‘…the major nutrient transporter, which as mentioned above, is transcribed and translated in the fat body and incorporated into developing oocytes.’

We have changed the wording as suggested by the reviewer (line 152).

Line 273 The authors say: ‘and is in turn regulated by this yolk protein’ Is the yolk protein Vg or amino acids not incorporated into Vg?

We have changed the wording to “is in turn regulated by amino acids not incorporated into this yolk protein“ (line 273-274).

Lines 322-326 and S4D Fig I think the authors should add triglyceride levels presented in Fig 4D to the S4D Fig for better comparison with other lipids.

Triglycerides are a subclass of neutral lipids, so they are already incorporated into figure S4D as part of the “Neutral lipids” columns. We have added a clause specifying that triglycerides are a subclass of neutral lipids: “some minor lipid classes (phospholipids and glycerophospholipids) were decreased (S4D Fig), while we detected a remarkable increase in triglycerides, a subclass of neutral lipids, (Fig 4D)” (line 325).

Lines 347-348 add reference: ovaries are degenerating and cannot accumulate further yolk (S1D Fig)

We have added a reference to the figure (line 348).

Line 351 word ‘overload’ might be too strong given the fact the level of triglycerides is about 1.2x-1.3x higher in Vg-depleted females compared to controls.

We have changed the word “overload” to “surplus” (line 351).

Finally, I wish you a Happy Holidays and a peaceful and prosperous New Year!

We hope that you had a restful break and we wish you a wonderful New Year!

**Have all data underlying the figures and results presented in the manuscript been provided?**
Large-scale datasets should be made available via a public repository as described in the *PLOS Genetics* [data availability policy](http://journals.plos.org/plosgenetics/s/data-availability), and numerical data that underlies graphs or summary statistics should be provided in spreadsheet form as supporting information.

Reviewer #1: Yes

Reviewer #2: Yes

Reviewer #3: Yes

PLOS authors have the option to publish the peer review history of their article ([what does this mean?](https://journals.plos.org/plosgenetics/s/editorial-and-peer-review-process#loc-peer-review-history)). If published, this will include your full peer review and any attached files.


If you choose “no”, your identity will remain anonymous but your review may still be made public.


**Do you want your identity to be public for this peer review?** For information about this choice, including consent withdrawal, please see our [Privacy Policy](https://www.plos.org/privacy-policy).

Reviewer #1: No

Reviewer #2: No

Reviewer #3: No
